# Diagnostic and treatment delay in women with cancer in pregnancy: a case-based study

**DOI:** 10.1007/s00404-026-08500-5

**Published:** 2026-07-01

**Authors:** Lillian Sun Liang, Iben Katinka Greiber, Mona Aarenstrup Karlsen, Berit Woetmann Pedersen, Birgitte Bruun Nielsen, Malene Kronborg Have, Rikke Bek Helmig, Maria Cathrine Schmidt, Jeanett Strandbygaard, Lone Storgaard

**Affiliations:** 1 Department of Gynecology and Obstetrics, Rigshospitalet, Copenhagen, Denmark; 2 Faculty of Health and Medical Sciences, University of Copenhagen, Copenhagen, Denmark; 3 Department of Obstetrics and Gynecology, Aarhus University Hospital, Aarhus, Denmark

**Keywords:** CIP, Pregnancy-associated cancer, PAC, Diagnostic delay, Treatment delay

## Abstract

**Purpose:**

More advanced stages at diagnosis and lower survival rates are seen in women diagnosed with cancer during pregnancy compared to non-pregnant women with similar cancers. This study aims to investigate diagnostic and treatment delays, cancer stage, and survival in Danish pregnant cancer patients.

**Methods:**

Patients from the Danish cancer in pregnancy database were included. Cancer diagnosis, treatment, and obstetrical data were collected from the database. Time to treatment was compared to national cancer patient pathways. Cancer stages at diagnosis were compared to epidemiological studies identified through a systematic literature search. Survival was compared to data from NORDCAN, a Nordic cancer statistics database.

**Results:**

The study included 74 patients diagnosed with cancer in pregnancy. The median total delay was 43.5 days (IQR 27.75–94.75) with a patient delay of three days (IQR 0–31), a diagnostic delay of 15 days (IQR 8–35), and a treatment delay of 9.5 days (IQR 0–19). Time from cancer suspicion to start of treatment fell within recommended national cancer patient pathway intervals in 80.0% of cases. Breast cancer and melanoma in pregnancy tended to have more advanced stages at diagnosis compared to non-pregnant patients. The 1- and 5-year mortality rates were 5.4% and 17.6%, respectively.

**Conclusion:**

Our findings suggest that pregnant cancer patients presented with advanced stages at diagnosis and lower survival rates compared to non-pregnant patients with similar cancers. Our results indicate that the main contributor to the delay in cancer in pregnancy in Denmark is delayed suspicion of cancer.

## What does this study add to the clinical work

**Table:** 

Diagnostic delay appears to be a major contributor to the more advanced cancer stage at diagnosis and the higher mortality observed in pregnant women with cancer. Cancer should be considered in pregnant women presenting with severe, persistent, or atypical pregnancy-related symptoms to facilitate earlier diagnosis and treatment.	

## Introduction

Cancer in pregnancy (CIP) is defined as cancer during pregnancy or diagnosed within 1 year postpartum. It affects one in 1000 pregnancies [1], with an incidence of one in 4000 when only accounting for cancer diagnosed during pregnancy [2]. CIP is a rare and complex condition, considering the mother’s need for treatment while balancing the health and development of the fetus. A Swedish register-based study found a 3.0% yearly increase in the incidence of cancer diagnosed during pregnancy between 1990 and 2017 [3].

Studies have shown more advanced stages of cancer at diagnosis and higher mortality rates in CIP patients compared to non-pregnant women with similar cancers. A delay in diagnosis and initiation of treatment is suspected to be a major cause [4–8].

**Fig. 1:**
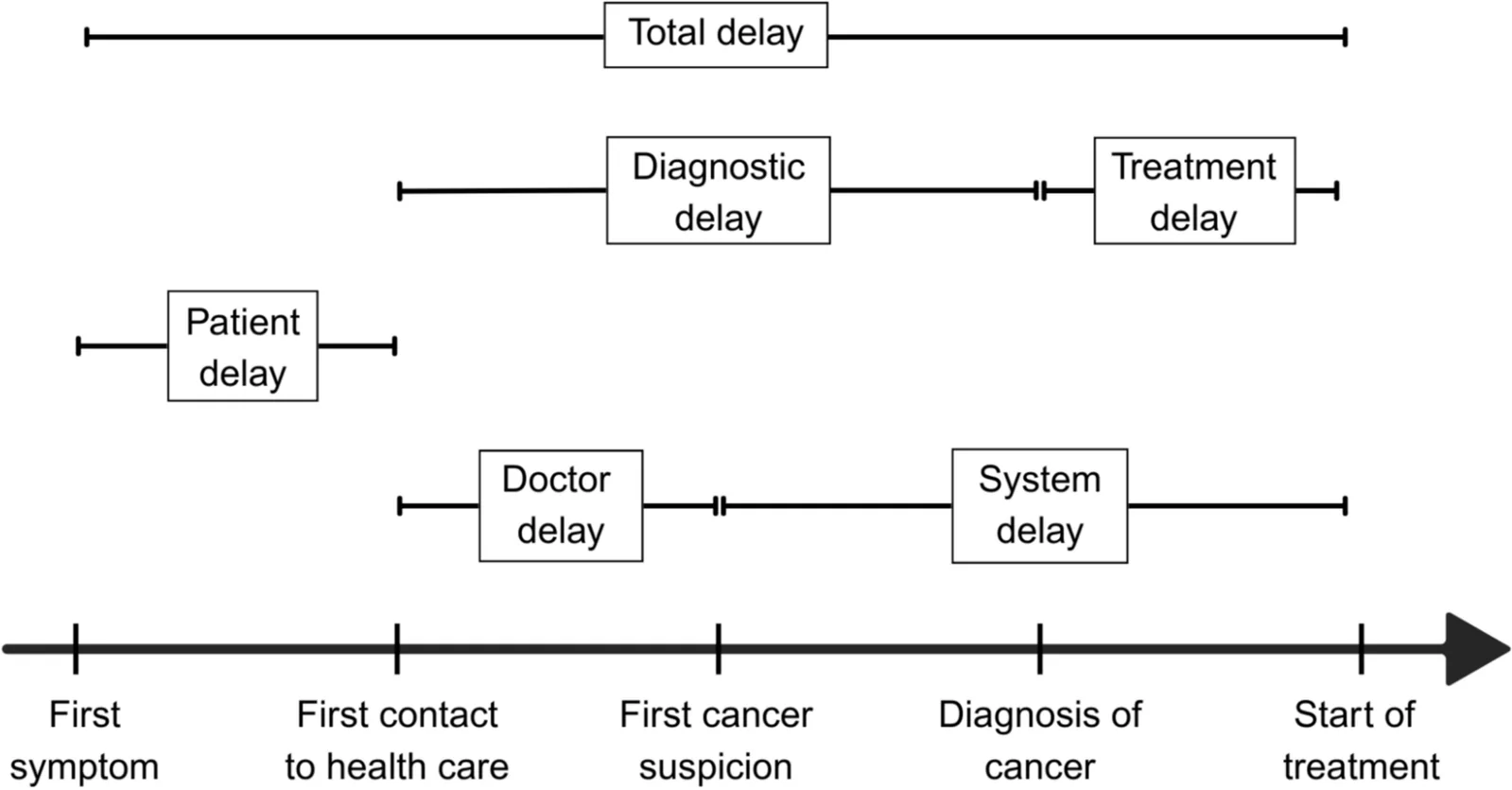
Overview of delay types in diagnosing and treating cancers used in this study on the patient’s timeline, from the date of the first symptom of cancer until the date of the start of cancer treatment

Delay can be divided into: patient, diagnostic, and treatment delay*.* The period after the patient’s first contact with the healthcare system can also be divided into doctor delay and system delay [9]. See Fig. 1 for an overview of delay types.

In 2008, national guidelines for fast-tracked cancer patient pathways (CPPs) were established in Denmark to minimize system delay and ensure diagnosis and treatment of suspected cancers within a recommended timeframe [10]. Recommendations cover the time from referral to CPP to the start of treatment and differ between cancers and treatments [11].

This study aims to investigate diagnostic and treatment delays in Danish CIP patients limited to patients diagnosed during pregnancy. Delays will be assessed in terms of time intervals, cancer stage at diagnosis, and survival.

## Methods

### Identification of the study population (CIP database)

The Danish CIP database consists of national, prospectively collected cases of pregnant women with active cancer from 2017 and is still ongoing. In Denmark, the management of CIP is centralized at three highly specialized university hospitals, from which inclusion in the database is coordinated. The database currently counts 84 patients and includes socio-demographic and cancer characteristics, diagnostic routes, cancer treatments, obstetric history, fetal scans, and mode of delivery. The database is continuously updated. The data for this study were collected in December 2025.

This study included patients with cancer (primary or relapse) diagnosed during pregnancy. For patients diagnosed with cancer relapse, symptoms, diagnosis, and the initiation of treatment were defined regarding the relapse. If fewer than three patients were diagnosed with a specific type of cancer, the cancer was grouped with similar cancer types based on anatomical location or cell type. The rarer cancer types were grouped in ‘other’ if no similar cancers were identified.

Pregnant women in Denmark have their due date estimated at a first-trimester scan at gestational week 11–13. Gestational age at diagnosis and delivery was calculated based on this estimated due date. In case of termination or miscarriage before the first-trimester scan, an early scan or the first day of the last menstrual period was used.

### Study design

This study is a case-based study based on the Danish CIP database.

Information from the database was compared to that of non-pregnant Danish cancer patients:

1. Time to treatment: Relevant CPP for each cancer type in this study was retrieved from the Danish Health Authority [11]. National data regarding time intervals and the proportion of cancer patients diagnosed within the recommended timeframe were found on Sundhedsdatabank.dk [12].
2. Stage at diagnosis: Stage and tumor characteristics at time of diagnosis for Danish women under 50 years old or premenopausal diagnosed after 2008 were found through a systematic search conducted in PubMed. The search was done for each type of cancer in March 2025. The search strings can be found in Appendix 1.
3. Survival rate: Age-specific survival rates for Danish women with cancer were found in NORDCAN [13], a database containing cancer statistics from the Nordic countries.

### Measures of outcome

Regarding time to diagnosis and treatment, five intervals were investigated:

1. *Patient delay* was calculated as the date of first contact with healthcare minus the date of the self-reported first symptom. If the date of the first symptom was registered as start, middle, or end of the month, the exact date was registered as 1st, 15th, or last day of the month, respectively. If no exact date had been given, the 1st of the month was registered. If a patient did not display symptoms before contacting the healthcare system, the patient delay was registered as zero.
2. *Diagnostic delay* was calculated as the date of diagnosis minus the date of first contact with healthcare.
3. *Treatment delay* was calculated as the start date of cancer treatment minus the date of diagnosis. If treatment was started before diagnosis, the treatment delay was registered as zero.
4. *Total delay* was calculated as the sum of patient, diagnostic, and treatment delay.
5. *The recommended interval from the CPPs* was found for the CIP patients referred to the CPPs. It was calculated as the start date of treatment minus the date of referral.

Patients with missing data for a given calculation were excluded from that specific analysis.

For the deceased patients, the time of survival was calculated as the date of death minus the date of diagnosis.

Follow-up time was calculated as the date of last follow-up minus the date of diagnosis.

### Statistical analysis

Statistics were done in Excel (version 2503 build 16.0.18623.20208) and R (version 4.4.3). Categorical variables were reported as numbers and percentages, and continuous variables were reported as median and interquartile range (IQR). Fisher’s exact test was used in Tables 4 and 5.

## Results

### Study group characteristics

Of the 84 patients from the CIP database, 10 did not meet the inclusion criteria for this study, because their cancer was diagnosed before pregnancy.

Of the 74 patients included, 5 (6.8%) were diagnosed with cancer relapse.

The patients were diagnosed with cancer throughout the entire pregnancy, with a median gestational age at diagnosis of 27 + 6 (IQR 15 + 2–34 + 0). Most (n = 68, 91.9%) pregnancies resulted in live births, of which 46 (67.6%) were delivered vaginally. Detailed patient characteristics are provided in Table 1.

**Table 1:** Characteristics of included patients from the Danish cancer in pregnancy database

Characteristics	Cancer in pregnancy database
Cases, no	74
Age (years), median (IQR)	34 (30–38)
BMI, median (IQR)	23.9 (21.6–27.0)^a^
Parity, No. (%)	
P0	31 (41.9)
P +	43 (58.1)
Gestational age at diagnosis, median (IQR)	27 + 6 (15 + 2—34 + 0)
Gestational age at delivery, median (IQR)	37 + 0 (35 + 4—38 + 3)
Mode of delivery, no. (%)	
Live births	68 (91.9)
Cesarean section	22 (32.4 of live births)
Elective cesarean section	18
Cancer indication	7
Obstetrical indication	8
Other indication	3
Emergency cesarean section	4
Vaginal	46 (67.6 of live births)
Spontaneous	16
Induced	30
Cancer indication	23
Obstetrical indication	5
Other indication	2
Miscarriage	2 (2.7)
Termination before gestational week 20	1 (1.4)
Cancer indication	1
Other^b^	3 (4.1)
First symptom, No. (%)	
Lumps	35 (47.3)
Pains and headaches	14 (18.9)
Changes in skin	12 (16.2)
Gastrointestinal symptoms	7 (9.5)
B-symptoms^c^	6 (8.1)
Fatigue	5 (6.8)
Respiratory symptoms	4 (5.4)
Other symptoms	14 (18.9)

*P0* Nulliparous, *P* + Multiparous

^a^Two patients with missing information regarding pre-pregnancy weight were excluded from the calculation

^b^One patient died before giving birth, two patients still pregnant

^c^B-symptoms include malaise, weight loss, and night sweats

This study included 17 different types of cancer, which were grouped into nine groups, with breast cancer and melanoma being the largest (see Table 2). Hematological cancers include lymphomas and leukemias. Head and neck cancers include thyroid cancers and squamous cell carcinomas. Cancers of the central nervous system (CNS) include astrocytomas and glioblastomas. Urogenital cancers include renal, cervical, ovarian, and vulvar cancers.

**Table 2:** Number of patients, stage at diagnosis, and delay intervals for the eight cancer groups and all patients included in this study from the CIP database

	Patients, no. (%)	Stage at diagnosis, no. (%)				Patient delay (days), median (IQR)	Diagnostic delay (days), median (IQR)	Treatment delay (days), median (IQR)	Total delay (days), median (IQR)
		Localized	Regional	Distant	Missing/not staged				
Breast	31 (41.9)	14 (45.2)	11 (35.5)	6 (19.4)	0 (0.0)	2 (0–17)^a^	10 (3.75–18.8)^b^	11 (6.5–20)	36 (24–54)^a^
Melanoma	12 (16.2)	9 (75.0)	2 (16.7)	1 (8.3)	0 (0.0)	1.5 (0–74.5)	12 (10–25.8)	0 (0–2.25)	66.5 (27.2–105)
Hematological	8 (10.8)	2 (25.0)	3 (37.5)	0 (0.0)	3 (37.5)	4 (0.75–16)	11 (5.5–35.8)	7.5 (2.75–13.2)	45 (20.8–52)
Head and neck (incl. thyroid)	7 (9.5)	6 (85.7)	1 (14.3)	0 (0.0)	0 (0.0)	31 (5–139)	19 (11.5–79.5)	15 (4.5–16.5)	63 (43.5–218)
Urogenital	5 (6.8)	3 (60.0)	1 (20.0)	1 (20.0)	0 (0.0)	18 (1–59)	74 (28–105)	6 (0–11)	112 (98–165)
Sarcomas	3 (4.0)	2 (66.7)	0 (0.0)	1 (33.3)	0 (0.0)	0 (0–369)	47 (41–519)	80 (44.5–120)	865 (454–1008)
Colon	3 (4.0)	1 (33.3)	0 (0.0)	2 (66.7)	0 (0.0)	2 (1–17)	42 (26.5–43)	0 (0–1)	45 (43.5–45.5)
Central nervous system	3 (4.0)	3 (100.0)	0 (0.0)	0 (0.0)	(0.0)	7 (5–17.5)	12 (6.5–19)	38 (20–86)	92 (49–122)
Other	2 (2.7)	0 (0.0)	0 (0.0)	2 (100.0)	0 (0.0)	9.5 (4.75–14.2)	25 (19–31)	10 (5–15)	44.5 (38.8–50.2)
All	74 (100.0)	40 (54.1)	18 (24.3)	13 (17.6)	3 (4.1)	3 (0–31)^a^	15 (8–35)^b^	9.5 (0–19)	43.5 (27.75–94.75)^a^

Cancer types were grouped with similar cancer types if fewer than three cases of a cancer type were identified in the CIP database. If no similar cancer types were identified, it would be grouped in ‘other’. Patient delay: time from first symptom to first contact with healthcare. Diagnostic delay: time from first contact with healthcare to diagnosis. Treatment delay: time from diagnosis to start of treatment. Total delay: sum of patient, diagnostic, and treatment delay

^a^Two patients were excluded from the calculation due to missing information

^b^One patient was excluded from the calculation due to missing information

### Delay intervals

For the analysis of patient delay, 72 patients were included. Two patients were excluded due to missing data regarding symptom onset. For the analysis of diagnostic delay, 73 patients were included. One patient was excluded due to missing data on the first healthcare contact. Therefore, 72 patients were included in the analysis of total delay.

Patient delay was registered as zero for seven patients, as they sought medical attention for other reasons before the onset of symptoms, or their cancers were discovered incidentally or during routine check-ups. Likewise, the treatment delay was registered as zero for 15 patients, since they received diagnostic surgery, or treatment was started before the final diagnosis.

For all types of cancer, the median total delay was 43.5 days (IQR 27.75–94.75). The median patient delay was 3 days (IQR 0–31), the median diagnostic delay was 15 days (IQR 8–35), and the median treatment delay was 9.5 days (IQR 0–19).

When separated into cancer groups, sarcomas displayed the longest median total delay and median treatment delay, but the shortest median patient delay. The longest median patient delay was found in head and neck cancers, and the longest diagnostic delay was found in urogenital cancers. The delays for the specific groups can be found in Table 2.

### Cancer patient pathways

The cancer types in this study were covered by 15 cancer patient pathways (CPPs) [11]. In this study, 55 patients were included in the analyses regarding the CPPs. Due to missing information on the date of referral or because they were not referred to the CPPs, 19 patients were excluded. Notably, none of the patients with colon cancer, CNS cancers, or cancers in the ‘*other*’ group were referred via these pathways. Of the included patients, 44 (80.0%) were diagnosed and treated within the recommended time of the CPPs. Intervals and fulfillment of the CPPs for the CIP patients, as well as the recommended intervals, are displayed in Table 3.

**Table 3:** Time to diagnosis and treatment for patients from the Danish Cancer in Pregnancy database and national data regarding the cancer patient pathways

	Cancer in pregnancy database			Recommended time intervals according to CPPs in days	Danish patients treated within recommended time, % (3rd, 2025)
	Patients referred to CPPs, no. (%)	Days from referral to start of treatment, median (IQR)	Patients treated within recommended time, no. (%)		
Breast	26 (83.9)	21.5 (14.75–33)	17 (65.4)	27–34	57–70
Melanoma	12 (100.0)	2 (0–8.25)	12 (100.0)	31	91
Hematological	5 (62.5)	13 (10–23)	5 (100.0)	6–43	93–94 ^a^
Head and neck (incl. thyroid)	7 (100.0)	9 (7–17)	7 (100.0)	28–39	62–87
Urogenital	2 (40.0)	15 (14–16)	2 (100.0)	28–53	55–94
Colon	0 (0.0)	–	–	37–41	71–83
Sarcomas	3 (100.0)	93 (31–187)	1 (33.3)	32–48	92 ^b^
Central nervous system	0 (0.0)	–	–		95
Other	0 (0.0)	–	–	41–57	76–90
ALL	55 (74.3)	16 (7–26)	44 (80.0)		

The cancer types identified in the CIP database were covered by 15 CPPs, which were grouped into nine cancer groups. The recommended time interval in the CPPs covers the time from referral to start of treatment, and varies according to cancer type and type of treatment

*CPPs* cancer patient pathways

^a^Statistics not available for acute myeloid leukemia

^b^Statistics not available for the 3rd quarter of 2025, substituted with 2nd quarter

### Stage at diagnosis

Apart from the ‘other’ cancer group, the colon cancer group displayed the highest proportion of distant disease (2/3, 67%). All five patients diagnosed with cancer relapse presented with metastatic disease. The stage at diagnosis for the different cancer groups is presented in Table 2.

From the systematic literature search, two studies regarding breast cancer and melanoma, respectively, were included:

Viuff et al. 2022 [14] compared survival for breast cancer patients with CIP (*n* = 134) and non-pregnant patients (*n* = 9112). Patients aged 15–44 diagnosed between 1973 and 2017 were included in this national, register-based study. The cancer characteristics of the 31 breast cancer patients from this study and the patients from Viuff et al. 2022 can be found in Table 4.

**Table 4:** Extent of breast cancer and tumor characteristics for the patients in the Danish Cancer in Pregnancy database compared to pregnant and non-pregnant patients from Viuff et al. 2022 [14]

	Cancer in pregnancy database	Viuff et al. 2022, pregnant (*P*)	Viuff et al. 2022, non-pregnant (*n*)	*P* value
Patients, No	31	134	9112	
Tumor size, No. (%)				
0–20 mm	6 (19.4)	52 (38.8)	4767 (52,3)	0.003 (*p*) < 0.001 (*n*)
21–50 mm	14 (45.2)	53 (39.6)	3198 (35,1)	
> 50 mm	9 (29.0)	9 (6.7)	507 (5,6)	
Missing	2 (6.5)	20 (14.9)	640 (7,0)	
Malignant lymph nodes, No. (%)				
0	12 (38.7)	50 (37.3)	4397 (48,3)	0.64 (*p*) 0.053 (*n*)
1–3	6 (19.4)	35 (26.1)	2736 (30,0)	
> 3	10 (32.3)	30 (22.4)	1424 (15,6)	
Missing	3 (9.7)	19 (14.2)	555 (6,1)	
Malignancy grade, No. (%)				
I	1 (3.2)	13 (9.7)	1828 (20,1)	0.49 (*p*) 0.004 (*n*)
II	8 (25.8)	40 (29.9)	3459 (38,0)	
III	14 (45.2)	57 (42.5)	2245 (24,6)	
Not graded	4 (12.9)	17 (12.7)	1109 (12,2)	
Missing	4 (12.9)	7 (5.2)	471 (5,2)	
Extent of breast cancer, No. (%)				
Localized	14 (45.2)	49 (36.6)	4405 (48,3)	0.011 (*p*) < 0.001 (*n*)
Regional	11 (35.5)	59 (44.0)	3955 (43,4)	
Distant	6 (19.4)	8 (6.0)	209 (2,3)	
Missing	0 (0.0)	18 (13.4)	542 (6,0)	

Bay et al. 2015 [15] reported the incidence and survival in Danish patients with melanoma from 1989 to 2011. The stage at diagnosis for women under 50 years of age diagnosed between 2009 and 2011 (*n* = 1300), and for the 12 melanoma patients from this study, can be found in Table 5.

**Table 5:** Melanoma stage at diagnosis for the patients in the Danish Cancer in Pregnancy database compared to women with melanoma under 50 years old in the period 2009–2011 from Bay et al. 2015 [15]

	Cancer in pregnancy database	Bay et al. 2015	*P* value
Patients, No	12	1300	
TNM stage, No. (%)			
I	5 (41.7)	1007 (77.5)	0.001
II	3 (25.0)	51 (3.9)	
III	2 (16.7)	52 (4.0)	
IV	1 (8.3)	16 (1.2)	
Missing	1 (8.3)	174 (13.4)	

### Survival

Of the included patients, 15 (20.3%) have passed away since diagnosis, with a median survival time of 26 months (IQR 8–44). Within the first year, four (5.4%) patients passed away, and within 5 years, 13 (17.6%) had passed away. Two deceased patients survived longer than 5 years. The cancer diagnoses for the deceased patients were distributed between breast (nine patients), colon (one), urogenital (two), lung (one), sarcoma (one), and CNS cancers (one). The median follow-up time was 32.5 months (IQR 16.75–58.5). In this study, 59 (79.7%) patients have a follow-up of 1 year or longer, and 18 (24.3%) have a follow-up of 5 years or longer.

## Discussion

### Delay intervals

In this study, the median total delay was 43.5 days. Among the delay components, the diagnostic delay had the longest median duration (15 days), whereas the patient delay had the shortest (3 days).

Patients with sarcomas had a long median total delay (865 days), while the median patient delay was 0 days, suggesting that the prolonged delay may reflect the known diagnostic challenges of sarcoma rather than pregnancy-related factors alone [16]. Sarcomas are treated with surgery and additional radiation therapy, which can be challenging during pregnancy, depending on cancer location and gestational age. Treatment might, therefore, have to be postponed until later in pregnancy or postpartum. For most cancer groups, the diagnostic delay was the main contributor to the total delay.

A recent UK study (*n* = 84) [17] on breast cancer in pregnancy found a median time from first symptoms to diagnosis of 3 weeks, which is longer than the 12 days for breast cancer patients in this study.

In Denmark, the management of CIP was centralized in 2015. Since 2004, there have been national guidelines for the management of breast cancer in pregnancy, and corresponding national guidelines for melanoma during pregnancy have subsequently been established [18]. In 2023, the International Advisory Board on Cancer in Pregnancy established a Danish advisory board [19, 20].

The centralization and national guidelines may contribute to the short delays experienced by patients with CIP in Denmark.

Cancer symptoms in pregnant women may be misinterpreted as pregnancy-related symptoms due to the overlap, such as nausea, constipation, and pain [8, 21]. These symptoms were also found in the study population. Likewise, diagnostic procedures may be inappropriately withheld due to concerns about potential harm to the fetus. Procedures like chest X-rays, low-dose CT scans, and MRIs are considered feasible in pregnancy with adequate indication [22].

Several studies have been conducted on delay intervals in Danish cancer patients [9, 23–27]. The studies were mainly conducted before the implementation of the CPPs. Since implementation, diagnostic delay has been reduced for patients diagnosed through the CPPs [28], making it difficult to compare diagnostic delay with this study. However, these studies report a median patient delay between 9 and 31 days, compared to three days found in this study. The short patient delay in CIP is likely due to heightened awareness of bodily changes or frequent contact with the healthcare system during pregnancy. This suggests that patient delay in CIP is not a major contributor to the total delay.

The doctor delay in this study was not calculated due to difficulties in estimating when the first cancer suspicion arose, even with information on referral to the CPPs. Many patients were referred to the CPPs after a diagnosis had been registered, possibly to ensure timely further investigation and treatment. This might be because no suspicion of cancer was present when performing the initial diagnostic procedure.

This is also reflected in the delays of patients with cancer relapse in pregnancy. They had a shorter patient and diagnostic delay, but similar treatment and total delay to the rest of the study group. This could indicate that both patient and doctor were more aware of the experienced symptoms being cancer symptoms.

### Cancer patient pathways

In the third quarter of 2025, the recommended time interval in the CPPs utilized in this study was fulfilled for 57–95% of cancer patients in Denmark [12]. For the patients in this study, the fulfillment of the recommended interval was 80%, meaning that the CIP patients do not have longer system delays than non-pregnant cancer patients. The only exception was for patients with sarcomas, of whom only one (33.3%) patient fulfilled the recommended interval, as seen in Table 3. None of the patients with colon or CNS cancer were referred via the CPPs, although the colon cancer group had the highest proportion of metastatic disease at diagnosis. These groups presented in most parts with gastrointestinal symptoms or generalized symptoms such as fatigue and headaches, which are typical pregnancy-related symptoms. This could imply that the main part of delay happens before suspicion of cancer, and that a major challenge in diagnosing CIP could be for healthcare professionals to suspect cancer. Studies show that a delayed suspicion of cancer by the physician might lead to a longer total delay [29–31] and that patients not diagnosed through the CPPs experience longer delays [28].

### Stage at diagnosis

Comparable studies reporting stage at diagnosis were only found for breast cancer and melanoma. [14, 15]. We found that the patients with CIP tended to have a more advanced stage at diagnosis compared to the non-pregnant patients (see Tables 4 and 5). Patients from this study with breast cancer tended to have large tumors compared to both the pregnant and non-pregnant patients from Viuff et al. Early stage melanomas are frequently managed locally and may not be referred to one of the three national centers. Consequently, the database tends to overrepresent advanced-stage cases.

### Survival

In Denmark, the 1- and 5-year survival rates for all cancer types combined among women aged 0–49 years were 96.8 and 89.7%, in the period 2018–2022 [13]. The 1- and 5-year survival rates in this study were 94.6% and 82.4%, respectively.

Since NORDCAN includes all patients diagnosed with cancer, the CIP population is also included. However, since the incidence of CIP is very small compared to the total incidence of cancer, the skew in data is expected to be minimal. Ideally, the comparison would be made with pregnant vs. non-pregnant patients, but this is not possible.

The mortality rate found in this study is equivalent to the one found in a UK study (*n* = 119) on CIP (21.3 vs. 20.2%) [32]. A big Swedish cohort study (*n* = 121,382) showed higher mortality for CIP patients with breast cancer and cancers of the upper digestive tract [33]. In this study, 60% of the deceased patients had breast cancer, and make up a majority of the overall mortality rate, even though the group only accounts for 40% of the study population.

### Strengths and limitations

This study is the first to report on diagnostic and treatment delays in Danish patients with CIP. The biggest strength of this study is the CIP database, which contains a thorough description of the course of diagnosis and treatment, making it possible to assess the different delays. Loss of follow-up was not a problem in this study, since the data used for calculations of delay and mortality rate were continuously registered and updated in the database.

However, due to the limited number of cases and short follow-up times in the Danish CIP database, it is difficult to perform meaningful statistical analyses. This must be kept in mind with the statistics performed in Tables 4 and 5. Selection bias could not be avoided in the study due to the voluntary inclusion in the CIP database, which relies on new patients being referred to the tertiary hospitals. Due to unawareness, referrals to tertiary centers do not always happen. This possibly leads to underestimation of specific patient groups, including patients with localized cancers treated with curative surgery, such as melanomas. Furthermore, some patients may decide to terminate their pregnancy soon after diagnosis of cancer and might not be referred to the CIP expert team at the tertiary hospitals for inclusion in the database.

## Conclusion

This study is the first to report on diagnostic and treatment delays in pregnant cancer patients in Denmark. Our findings suggest that pregnant patients presented with more advanced stages of cancer at the time of diagnosis and lower survival rates compared to non-pregnant women with similar cancers. This shows that delays occur in Danish patients with cancer in pregnancy. However, the time to diagnosis and treatment according to the cancer patient pathways does not differ from other cancer patients in Denmark. Therefore, our results indicate that the main contributor to the delay in cancer in pregnancy in Denmark is the delayed suspicion of cancer*.*

## Data Availability

No datasets were generated or analyzed during the current study.

## Appendix 1: Systematic literature search—search strings

### Breast cancer

(women[Text Word] OR woman[Text Word] OR female*[Text Word] OR "Women"[Mesh] OR "Female"[Mesh]) AND ("cancer stag*"[Text Word] OR "TNM"[Text Word] OR "tumor characteristic*"[Text Word] OR "tumour characteristic*"[Text Word] OR "stage at diagnosis"[Text Word] OR "Neoplasm Staging"[MeSH Terms] OR "descriptive"[Text Word]) AND (Denmark[Text Word] OR Danish[Text Word] OR "Denmark"[Mesh] OR DBCG [All Fields] OR "Danish breast cancer"[All Fields]) AND ("breast cancer"[Text Word] OR "mamma* cancer"[Text Word] OR "Breast Neoplasms"[MeSH Terms] OR "breast carcinoma"[Text Word]).

### Melanoma

(women[Text Word] OR woman[Text Word] OR female*[Text Word] OR "Women"[Mesh] OR "Female"[Mesh]) AND ("cancer stag*"[Text Word] OR "TNM"[Text Word] OR "tumor characteristic*"[Text Word] OR "tumour characteristic*"[Text Word] OR "stage at diagnosis"[Text Word] OR "Neoplasm Staging"[MeSH Terms] OR "descriptive"[Text Word]) AND (Denmark[Text Word] OR Danish[Text Word] OR "Denmark"[Mesh] OR "DMG"[All Fields] OR "Danish melanoma"[All Fields]) AND ("melanoma*"[Text Word] OR "Melanoma"[MeSH Terms]).

### Head and neck cancers

(women[Text Word] OR woman[Text Word] OR female*[Text Word] OR "Women"[Mesh] OR "Female"[Mesh]) AND (“cancer stag*”[Text Word] OR "TNM"[Text Word] OR "tumor characteristic*"[Text Word] OR "tumour characteristic*"[Text Word] OR "stage at diagnosis"[Text Word] OR "Neoplasm Staging"[MeSH Terms] OR "descriptive"[Text Word]) AND (Denmark[Text Word] OR Danish[Text Word] OR "Denmark"[Mesh] OR "DAHANCA"[All Fields] OR "Danish head and neck cancer"[All Fields]) AND ("head and neck cancer*"[Text Word] OR "thyroid cancer*"[Text Word] OR "Head and Neck Neoplasms"[MeSH Terms]).

### Hematological cancers

(women[Text Word] OR woman[Text Word] OR female*[Text Word] OR "Women"[Mesh] OR "Female"[Mesh]) AND ("cancer stag*"[Text Word] OR "TNM"[Text Word] OR "tumor characteristic*"[Text Word] OR "tumour characteristic*"[Text Word] OR "stage at diagnosis"[Text Word] OR "Neoplasm Staging"[MeSH Terms] OR "descriptive"[Text Word]) AND (Denmark[Text Word] OR Danish[Text Word] OR "Denmark"[Mesh] OR "ALG"[All Fields] OR "DLG"[All Fields] OR "Danish lymphoma"[All Fields] OR "Acute leukemia group"[All Fields]) AND ("Leukemia"[MeSH Terms] AND "Lymphoma"[MeSH Terms]) OR ("acute leukemia*"[Text Word] OR "lymphoma*"[Text Word]).

### Urogenital cancers

(women[Text Word] OR woman[Text Word] OR female*[Text Word] OR "Women"[Mesh] OR "Female"[Mesh]) AND (“cancer stag*”[Text Word] OR "TNM"[Text Word] OR "tumor characteristic*"[Text Word] OR "tumour characteristic*"[Text Word] OR "stage at diagnosis"[Text Word] OR "Neoplasm Staging"[MeSH Terms] OR "descriptive"[Text Word]) AND (Denmark[Text Word] OR Danish[Text Word] OR "Denmark"[Mesh] OR “DGCG”[All Fields] OR "Danish gynecological cancer"[All Fields]).

### Sarcomas

(women[Text Word] OR woman[Text Word] OR female*[Text Word] OR "Women"[Mesh] OR "Female"[Mesh]) AND ("cancer stag*"[Text Word] OR "TNM"[Text Word] OR "tumor characteristic*"[Text Word] OR "tumour characteristic*"[Text Word] OR "stage at diagnosis"[Text Word] OR "Neoplasm Staging"[MeSH Terms] OR "descriptive"[Text Word]) AND (Denmark[Text Word] OR Danish[Text Word] OR "Denmark"[Mesh] OR "DSG"[All Fields] OR "Danish Sarcoma"[All Fields]) AND ("Sarcoma"[Text Word] OR "Sarcoma"[MeSH Terms]).

### Colon cancer

(women[Text Word] OR woman[Text Word] OR female*[Text Word] OR "Women"[Mesh] OR "Female"[Mesh]) AND ("cancer stag*"[Text Word] OR "TNM"[Text Word] OR "tumor characteristic*"[Text Word] OR "tumour characteristic*"[Text Word] OR "stage at diagnosis"[Text Word] OR "Neoplasm Staging"[MeSH Terms] OR "descriptive"[Text Word]) AND (Denmark[Text Word] OR Danish[Text Word] OR "Denmark"[Mesh] OR "Danish Colorectal Cancer"[All Fields] OR "DCCG"[All Fields]) AND ("colon* cancer"[Text Word] OR "colon* neoplasm*"[Text Word] OR "colon* tumo*"[Text Word] OR "Colonic Neoplasms"[MeSH Terms]).

## Abbreviations

CIP: Cancer in pregnancy
CPP: Cancer patient pathway
IQR: Interquartile range
CNS: Central nervous system
